# The role of individual level health resources on health outcomes of newly settled migrants in Sweden

**DOI:** 10.1093/eurpub/ckac131.313

**Published:** 2022-10-25

**Authors:** M Al-Adhami, E Berglund, J Wångdahl, K Hedrick, R Salari

**Affiliations:** 1 SWEDESD, Department of Women’s and Children’s Health, Uppsala, Sweden; 2 CHAP, Department of Public Health & Nursing Science, Uppsala, Sweden; 3 Department of Public Health and Nursing Science, Uppsala, Sweden

## Abstract

Abstract citation ID ckac131.313 has been removed

